# Morphometric anatomy of the middle cranial fossa via the anterior petrosal (Kawase) approach: a bilateral cadaveric study

**DOI:** 10.3389/fsurg.2026.1780349

**Published:** 2026-06-23

**Authors:** Huong Dinh Nguyen, Tuyen Quang Le, Hung Manh Ngo, Hung Dinh Kieu

**Affiliations:** 1 Department of Surgery, Hanoi Medical University, Hanoi, Vietnam; 2 Neuroscience Center, Vinmec Times City International Hospital, Hanoi, Vietnam; 3 Department of Anatomy, Pham Ngoc Thach University of Medicine, Ho Chi Minh City, Vietnam; 4 Faculty of Medicine, University of Medicine and Pharmacy, Vietnam National University, Hanoi, Vietnam; 5 Department of Neurosurgery, Hospital of University of Medicine and Pharmacy—Linh Dam, Hanoi, Vietnam; 6 Department of Neurosurgery and Spine Surgery, Hanoi Medical University Hospital, Hanoi, Vietnam; 7 Vietlife Clinique System, Hanoi, Vietnam

**Keywords:** anterior petrosal approach, cadaveric study, middle cranial fossa, morphometry, skull base anatomy

## Abstract

**Background:**

The anterior petrosal (Kawase) approach is a well-established extradural corridor to the petroclival and upper clival regions; however, comprehensive bilateral morphometric data describing middle cranial fossa anatomy, particularly in Southeast Asian populations, remain limited.

**Objective:**

To provide a comprehensive morphometric description of the microsurgical anatomy of the middle cranial fossa through the anterior petrosal (Kawase) approach based on bilateral cadaveric dissections.

**Methods:**

A descriptive cadaveric study was performed on 21 formalin-fixed adult Vietnamese cadaveric heads (42 sides). Standardized extradural dissections following the anterior petrosal approach were conducted, and predefined linear and angular morphometric measurements of key osseous, neural, and vascular landmarks were obtained bilaterally.

**Results:**

The greater superficial petrosal nerve (GSPN), trigeminal ganglion, cochlea, internal auditory canal (IAC), superior semicircular canal, and petrous internal carotid artery were consistently identified in all specimens. The mean GSPN length was 10.66 ± 2.44 mm. Key inter-foraminal and neurovascular distances included a mean distance of 29.68 ± 1.97 mm from the foramen spinosum to the zygomatic root and 11.70 ± 2.44 mm between the foramen ovale and foramen rotundum. Cochlear-related measurements demonstrated mean distances of 3.39 ± 0.98 mm to the geniculate ganglion and 4.56 ± 1.29 mm to the internal carotid artery genu. Angular analysis showed a mean angle of 121.82 ± 15.38° between the GSPN and arcuate eminence, 104.07 ± 13.25° between the GSPN and superior semicircular canal, and 45.42 ± 12.09° between the IAC and superior semicircular canal. Paired right–left comparisons demonstrated overall bilateral symmetry across most linear and angular parameters, with a significant side-to-side difference observed only in the IAC–SSC angle.

**Conclusions:**

This study provides a comprehensive bilateral morphometric characterization of the middle cranial fossa via the anterior petrosal approach in a Vietnamese population. The findings confirm the reliability of key anatomical landmarks, delineate their spatial relationships, and demonstrate predominantly bilateral symmetry, thereby offering population-specific anatomical data that may support surgical planning, neuronavigation, and training in skull base surgery.

## Introduction

The middle cranial fossa is a densely populated skull base compartment that contains critical neurovascular structures and provides key operative corridors for lesions involving the petroclival region, Meckel's cave, the posterior cavernous sinus, and the upper clivus. Precise anatomical knowledge of the middle cranial fossa floor, including the spatial relationships among the foramen spinosum, foramen ovale, foramen rotundum, the greater superficial petrosal nerve (GSPN), and labyrinthine structures, is essential to avoid injury to the facial nerve, cochlea, internal carotid artery, and trigeminal system during extradural dissection and drilling. Contemporary surgical–anatomical reviews and microsurgical teaching resources consistently emphasize that safe skull base surgery depends on reproducible identification of these landmarks and an accurate understanding of their variability across individuals (1–3).

Among extradural routes to the petroclival and retrosellar/upper clival regions, the anterior petrosal approach (Kawase approach) has become a widely adopted technique. This approach is based on extradural drilling of the petrous apex within a defined anatomical corridor, commonly referred to as Kawase's rhomboid (or quadrangle), which is bounded laterally by the GSPN, medially by the trigeminal nerve, posteriorly by the arcuate eminence, and anteriorly by the petrous ridge. This geometric construct provides a clear and practical anatomical framework for anterior petrosectomy and forms the basis for the present morphometric analysis (2, 4, 5).

Previous anatomical and surgical studies have described individual landmarks relevant to this approach, including the GSPN, arcuate eminence, and various foramina, as well as techniques for localizing deeper structures such as the internal auditory canal (IAC) (3, 6, 7). However, these studies are heterogeneous in both scope and methodology, often focusing on selected landmarks or single measurement types rather than providing an integrated morphometric framework.

To address the limitations of relying on individual landmarks, several alternative localization strategies have been proposed, including geometric relationships between skull base foramina, angular reference systems, and simplified landmark-based rules. While these approaches have improved intraoperative orientation, they also underscore the inherent variability of the petrous bone and the absence of universally reliable reference systems (8–14).

Importantly, several key gaps remain in the current literature. First, many studies rely on unilateral or pooled data, limiting the assessment of bilateral symmetry, which is clinically relevant for surgical orientation. Second, quantitative analyses frequently focus on either linear or angular measurements in isolation, without integrating both into a unified spatial framework. Third, definitions of measurement parameters and anatomical reference points vary considerably across studies, reducing comparability. Finally, population-specific morphometric data—particularly from Southeast Asian cohorts—remain limited, despite known ethnic variation in skull base anatomy (3, 15, 16). Taken together, these limitations highlight the need for a structured morphometric approach that integrates bilateral data within a consistent anatomical framework, such as Kawase's rhomboid, to improve both reproducibility and clinical applicability.

To address these limitations, a structured and comprehensive morphometric analysis that integrates bilateral data, standardized measurement definitions, and clinically relevant anatomical relationships is needed. Such an approach may provide a more consistent anatomical reference for surgical planning and improve the safety of anterior petrosectomy. Therefore, we conducted a bilateral cadaveric study to provide a comprehensive morphometric description of the middle cranial fossa through the anterior petrosal (Kawase) approach, with particular emphasis on the spatial relationships among key osseous, neural, and vascular landmarks.

## Materials and methods

### Study design

This study was designed as a descriptive cadaveric morphometric analysis aimed at providing a comprehensive anatomical characterization of the middle cranial fossa through the anterior petrosal approach. Bilateral dissections were performed to evaluate linear, angular, and relational measurements of key neurovascular and osseous landmarks relevant to microsurgical skull base procedures.

### Specimens

A total of 21 formalin-fixed adult human cadaveric heads were included, yielding 42 middle cranial fossae for analysis. Specimens were obtained from the Departments of Anatomy at the University of Medicine and Pharmacy at Ho Chi Minh City and Pham Ngoc Thach University of Medicine. Information regarding the exact age at death of the cadavers was not available from institutional records at either contributing anatomy department. Cadavers with gross cranial base deformities, prior skull base surgery, or visible destructive pathology affecting the middle cranial fossa were excluded. All available sides meeting predefined inclusion criteria were analyzed independently.

### Dissection technique

All dissections were performed under surgical microscopy following a standardized protocol adapted from the classical anterior petrosal approach. After positioning the cadaveric head in a neutral supine position, a temporal craniotomy was simulated to allow extradural access to the middle cranial fossa. The temporal dura mater was carefully elevated from lateral to medial, preserving the integrity of underlying neurovascular structures.

**Figure 1:**
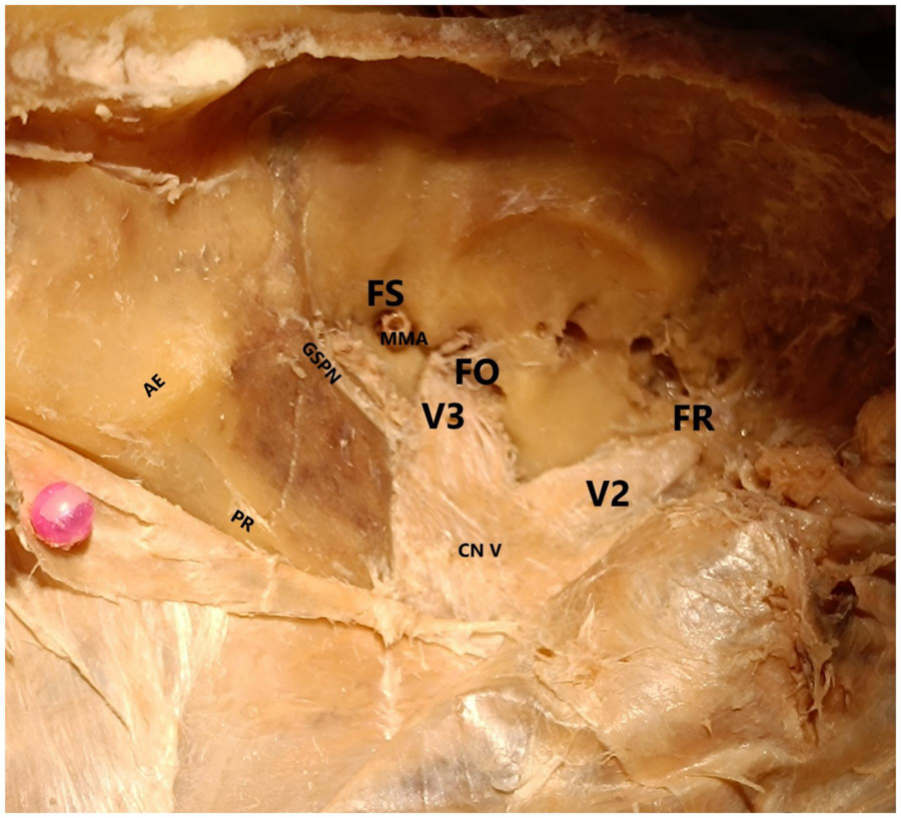
Left middle cranial fossa following extradural elevation of the temporal dura, prior to drilling, showing key osseous and neural landmarks. FS, foramen spinosum; FO, foramen ovale; FR, foramen rotundum; GSPN, greater superficial petrosal nerve; AE, arcuate eminence; CNV, trigeminal nerve (V2, maxillary division; V3, mandibular division); MMA, middle meningeal artery; PR, petrous ridge.

Initial extradural landmarks were identified, including the middle meningeal artery, foramen spinosum, and foramen ovale, which served as reference points for subsequent exposure. Representative extradural exposure prior to drilling, with identification of the foramen spinosum (FS), foramen ovale (FO), foramen rotundum (FR), greater superficial petrosal nerve (GSPN), arcuate eminence (AE), and trigeminal nerve branches (V2, V3), is shown in Figure 1. The GSPN was identified coursing anteromedially from the geniculate ganglion toward the foramen lacerum and was meticulously skeletonized along its visible length. The arcuate eminence was exposed as a surface landmark corresponding to the superior semicircular canal.

**Figure 2:**
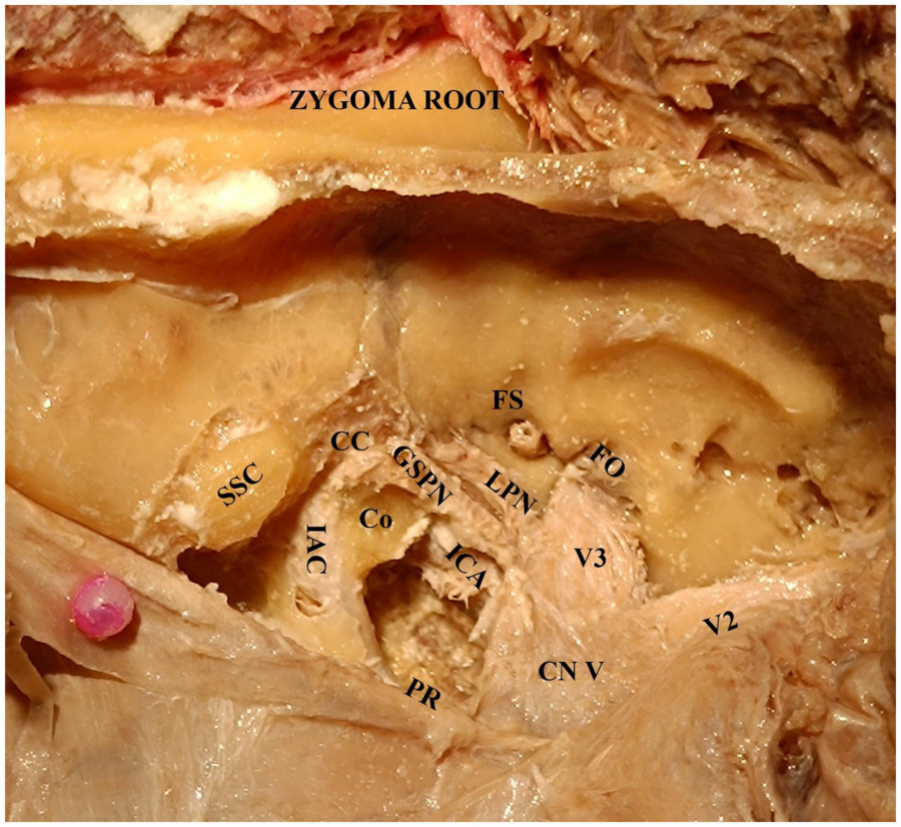
Left middle cranial fossa after completion of Kawase quadrangle drilling, demonstrating key deep surgical landmarks. FS, foramen spinosum; FO, foramen ovale; GSPN, greater superficial petrosal nerve; LPN, lesser petrosal nerve; CN V, trigeminal nerve (V2, V3); ICA, internal carotid artery (petrous segment); SSC, superior semicircular canal; GG, geniculate ganglion; Co, cochlea; PR, petrous ridge.

Progressive extradural drilling was performed when necessary to delineate the boundaries of Kawase's quadrangle, defined by the GSPN laterally, the trigeminal nerve medially, the arcuate eminence posteriorly, and the petrous ridge anteriorly. Deeper landmarks, including the geniculate ganglion, IAC, cochlea, superior semicircular canal, and the petrous segment of the internal carotid artery, were identified through careful stepwise bone removal while maintaining anatomical orientation. The completed Kawase quadrangle drilling and the resulting deep surgical exposure (including the petrous ICA, superior semicircular canal, geniculate ganglion, and cochlea) are illustrated in Figure 2.

Special attention was paid to the identification and preservation of cranial nerves IV, V, VI, VII, and VIII, as well as vascular structures encountered within the operative corridor, including the anterior inferior cerebellar artery (AICA) when visible. All dissections and anatomical identifications were performed by a single board-certified neurosurgeon experienced in skull base surgery, ensuring consistency of technique and minimizing inter-observer variability across specimens.

### Morphometric measurements

**Figure 3:**
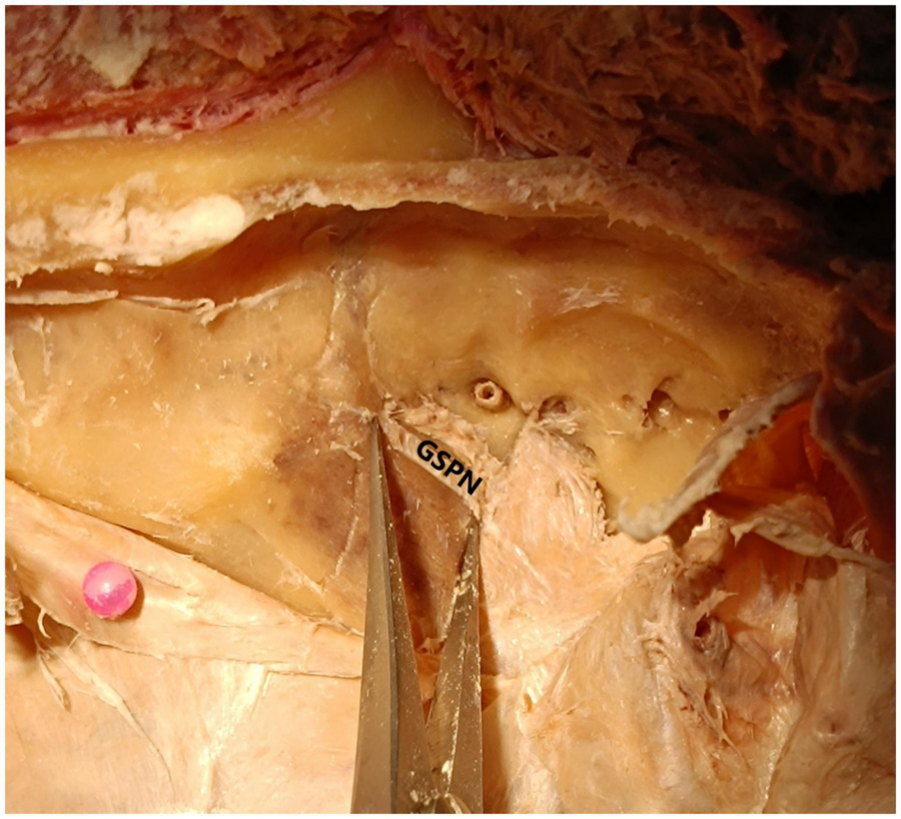
Representative linear measurement technique for GSPN intracranial length using calibrated digital calipers. Measurement extends from the geniculate ganglion to the intersection of the GSPN with the trigeminal nerve axis. GSPN, greater superficial petrosal nerve.

Morphometric measurements were predefined prior to dissection and focused on anatomical relationships of direct relevance to the anterior petrosal approach. Linear measurements were obtained using calibrated digital calipers and recorded in millimeters, while angular measurements were recorded in degrees using standardized geometric reference planes derived from identifiable bony and neural landmarks. A representative linear measurement setup (GSPN intracranial length measured from the geniculate ganglion to the intersection of the GSPN with the trigeminal nerve axis using calibrated digital calipers) is shown in Figure 3.

**Figure 4:**
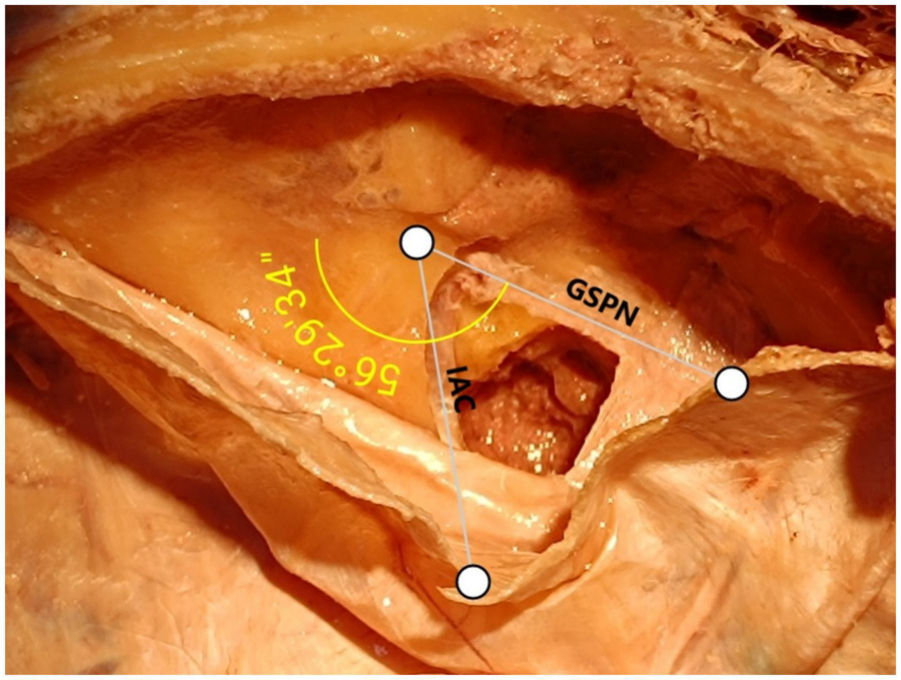
Representative angular measurement technique illustrating the GSPN–IAC angle after full extradural exposure. The angle is defined between the longitudinal axes of the GSPN and the internal acoustic canal (IAC). GSPN, greater superficial petrosal nerve; IAC, internal acoustic canal.

All linear distances were measured as straight-line Euclidean distances between the nearest predefined landmarks in the horizontal plane (parallel to the middle cranial fossa floor), unless otherwise specified. All angles were measured in the horizontal plane (parallel to the middle cranial fossa floor), and the axis of each structure was defined according to prespecified anatomical landmarks. A representative angular measurement technique is shown in Figure 4, in which the GSPN–IAC angle is defined by the intersection of the longitudinal axis of the GSPN and the longitudinal axis of the internal acoustic canal after full extradural exposure.

Linear parameters included nerve lengths, inter-foraminal distances, and spatial relationships between the GSPN, trigeminal nerve, cochlea, internal carotid artery, IAC, and superior semicircular canal. Angular measurements assessed the orientation of the GSPN relative to the arcuate eminence, superior semicircular canal, and IAC, as well as the angular relationship between the IAC and the superior semicircular canal. Each measurement was performed with the specimen positioned in a reproducible orientation to reduce measurement bias. Representative linear and angular measurement methods were documented to ensure reproducibility and to clarify anatomical reference points.

To ensure reproducibility, the following operational definitions were applied for key variables used in the final analysis:

- ZGR–FS distance: from the posterior point of the zygomatic root to the outer margin of the foramen spinosum.
- FS–FO distance: shortest bony distance between the posterior margin of the foramen ovale and the anterior margin of the foramen spinosum.
- FO–FR distance: shortest bony distance between the foramen ovale and foramen rotundum margins.
- GSPN length (surface segment): from P1 (greater petrosal nerve hiatus) to P2 (intersection with V3), measured along the nerve course.
- GG–GSPN (true GSPN) length: from the geniculate ganglion to the GSPN–V3 intersection.
- GSPN–ICA distance: shortest distance from the medial border of the GSPN to the medial border of the horizontal petrous ICA.
- V–IAC distance: from the posterior border of the trigeminal nerve to the anterior border of the internal acoustic canal.
- IAC–SSC distance: shortest distance from the IAC to the SSC at the nearest point.
- Cochlea–GG distance: from the center of the basal turn of the cochlea to the center of the geniculate ganglion.
- Cochlea–GSPN distance: shortest distance from the cochlear basal turn center to the GSPN–V3 intersection.
- Cochlea–ICA genu distance: shortest distance from the cochlear basal turn center to the medial wall of the ICA genu.
- Cochlea–SSC distance: shortest distance from the cochlear basal turn center to the midpoint of the SSC.
- GG–SSC distance: shortest distance from the geniculate ganglion center to the midpoint of the SSC.
- ICA horizontal length: measured along the actual course of the horizontal petrous ICA from the genu to the point where it turns toward the cavernous sinus.

Angular variables were defined as follows:

- GSPN–AE angle: angle between the GSPN axis (P1→P2) and the long axis of the arcuate eminence.
- GSPN–SSC angle: angle between the GSPN axis and the SSC axis (line connecting the two limbs of the canal at the vestibular segment).
- GSPN–IAC angle: angle between the GSPN axis and the IAC axis (from the center of the anterior IAC margin to the fundus center).
- IAC–SSC angle: angle between the IAC axis and the SSC axis

Measurements were recorded only when the anatomical structures involved could be clearly and confidently identified. When visualization was insufficient due to anatomical variation, preservation-related factors, or limited exposure inherent to the approach, the measurement was designated as missing and explicitly documented. No estimation or interpolation of missing values was performed.

Each side of the middle cranial fossa was treated as an independent observational unit for descriptive analyses. For variables available on both sides of the same specimen, paired right–left measurements were recorded to allow subsequent side-to-side comparisons. All measurements were documented immediately after acquisition using standardized data collection forms to minimize transcription errors. All dissections and measurements were performed by a single experienced neurosurgeon following a standardized protocol with predefined landmarks to ensure procedural consistency; however, formal intra-observer reliability testing was not conducted and is acknowledged as a limitation.

### Data analysis

All data were entered into a Microsoft Excel spreadsheet and subsequently analyzed using Stata version 17 (StataCorp, College Station, TX, USA). Descriptive statistics were calculated using available observations only, with continuous variables summarized as mean ± standard deviation or median with interquartile range, depending on data distribution; missing values were explicitly reported and no data imputation was performed. Categorical variables were presented as frequencies and percentages. For side-stratified comparisons, measurements from right and left sides were treated as independent observations and compared using independent-samples t-tests. For paired right–left comparisons, analyses were restricted to specimens with bilateral data, and paired t-tests were applied. Sex-stratified comparisons were performed using independent-samples t-tests. All tests were two-sided, and a *p*-value < 0.05 was considered statistically significant.

## Results

### Specimen characteristics and landmark identification

A total of 42 middle cranial fossa sides from 21 cadaveric heads were analyzed. The mean age of the cadavers was 69.95 ± 12.48 years. The sample included 26 male sides and 16 female sides, with an equal right–left distribution (21 sides each). Table 1 summarizes the general characteristics and morphological features stratified by side. The GSPN, trigeminal ganglion, cochlea, IAC, superior semicircular canal, and petrous ICA were identified in all dissections (100%). The arcuate eminence was also identifiable in all specimens, with variable morphological configurations (Table 2).

**Table 1:** General characteristics and morphological features of the examined specimens, stratified by side (*N* = 42 sides).

Variable	Total (*N* = 42)	Right (*N* = 21)	Left (*N* = 21)
	Count (% of total)	Count (% of total)	Count (% of total)
Specimen source			
PNT	22 (52.4%)	11 (52.4%)	11 (52.4%)
DHYD	20 (47.6%)	10 (47.6%)	10 (47.6%)
Sex			
Male	26 (61.9%)	13 (61.9%)	13 (61.9%)
Female	16 (38.1%)	8 (38.1%)	8 (38.1%)
Foramen spinosum morphology			
Oval	24 (57.1%)	12 (57.1%)	12 (57.1%)
Round	13 (31.0%)	6 (28.6%)	7 (33,3%)
Undefined	5 (11.9%)	3 (14.3%)	2 (9.5%)
Foramen ovale morphology			
Oval	32 (76.2%)	17 (81.0%)	15 (71.4%)
Round	8 (19.0%)	3 (14.3%)	5 (23.8%)
Undefined	2 (4.8%)	1 (4.8%)	1 (4.8%)
Foramen rotundum morphology			
Round	42 (100%)	21 (100%)	21 (100%)
GSPN coverage status			
Not covered	24 (57.1%)	12 (57.1%)	12 (57.1%)
Covered	18 (42.9%)	9 (42.9%)	9 (42.9%)
Geniculate ganglion exposure			
Not exposed	35 (83.3%)	19 (90.5%)	16 (76.2%)
Exposed	7 (16.7%)	2 (9.5%)	5 (23.8%)
Lesser petrosal nerve presence			
Present	30 (71.4%)	13 (61.9%)	17 (81.0%)
Absent	12 (28.6%)	8 (38.1%)	4 (19.0%)

Data are presented as *n* (%). PNT, pham ngoc thach university of medicine; UMP, university of medicine and pharmacy at Ho Chi Minh City; GSPN, greater superficial petrosal nerve.

**Table 2:** Morphological classification of the arcuate eminence and its relationship with the superior semicircular canal, stratified by side (*N* = 42 sides).

Variable	Total (*N* = 42)	Right (*N* = 21)	Left (*N* = 21)
	Count (% of total)	Count (% of total)	Count (% of total)
AE morphological configuration			
Straight flat	5 (11.9%)	2 (9.5%)	3 (14.3%)
Straight convex	17 (40.5%)	8 (38.1%)	9 (42.9%)
Spherical	2 (4.8%)	2 (9.5%)	0 (0%)
Single arch	8 (19.0%)	3 (14.3%)	5 (23.8%)
Double arch	7 (16.7%)	5 (23.8%)	2 (9.5%)
Complex arch	3 (7.1%)	1 (4.8%)	2 (9.5%)
AE–SSC anatomical relationship			
Not overlapping	14 (33.3%)	8 (38.1%)	6 (28.6%)
Completely overlapping	13 (31.0%)	4 (19.0%)	9 (42.9%)
Partially overlapping	15 (35.7%)	9 (42.9%)	6 (28.6%)

Data are presented as n (%). AE, arcuate eminence; SSC, superior semicircular canal.

### Arcuate eminence morphology

The arcuate eminence (AE) showed considerable morphological variability across specimens (Table 2). The straight convex type was the most common configuration (40.5%), followed by single arch (19.0%) and double arch (16.7%). Less frequent patterns included straight flat (11.9%), complex arch (7.1%), and spherical forms (4.8%). Regarding the relationship between the AE and the superior semicircular canal (SSC), partial overlap was observed most frequently (35.7%), followed by no overlap (33.3%) and complete overlap (31.0%). No significant differences were observed between the right and left sides.

### Linear morphometric measurements

Linear morphometric data for all 42 sides are summarized in Table 3, with separate values reported for the right and left sides. The mean GSPN length was 10.66 ± 2.44 mm (range, 6.01–16.80 mm). Key inter-foraminal distances included 29.68 ± 1.97 mm from the foramen spinosum to the zygomatic root and 11.70 ± 2.44 mm between the foramen ovale and foramen rotundum. The mean GSPN–ICA distance was 1.84 ± 1.52 mm (range, 0.00–5.65 mm), indicating close proximity of the GSPN to the petrous ICA in some specimens, with direct contact observed in 2 sides. Cochlear-related measurements showed mean distances of 3.39 ± 0.98 mm to the geniculate ganglion and 4.56 ± 1.29 mm to the ICA genu. The GG–GSPN (true GSPN) length was 13.78 ± 1.78 mm, and the GG–SSC distance was 8.82 ± 1.49 mm. Statistically significant right–left differences were identified for four parameters (*p* < 0.05): the GSPN–ICA distance (right 1.80 ± 1.78 mm vs. left 1.88 ± 1.26 mm, *p* = 0.0134), the cochlea–GSPN distance (right 11.63 ± 1.68 mm vs. left 11.74 ± 1.96 mm, *p* = 0.0148), the GG–GSPN (true GSPN) length (right 13.84 ± 1.79 mm vs. left 13.71 ± 1.81 mm, *p* = 0.0352), and the ICA horizontal length (right 6.78 ± 1.50 mm vs. left 6.54 ± 1.87 mm, *p* = 0.0173). All other linear measurements showed no statistically significant side differences.

**Table 3:** Linear morphometric measurements of key anatomical landmarks in the middle cranial fossa, stratified by side (mm).

Measurement	Total (*N* = 42)				Right (*N* = 21)	Left (*N* = 21)	*p*-value
	Mean ± SD	Median [IQR]	Min	Max	Mean ± SD	Mean ± SD	
ZGR–FS distance (mm)	29.68 ± 1.97	29.91 [28.45–30.87]	25.37	33.80	29.38 ± 1.76	29.99 ± 2.16	0.8499
FS–FO distance (mm)	3.08 ± 0.92	2.94 [2.41–3.52]	1.29	5.40	3.26 ± 1.00	2.88 ± 0.82	0.1498
FO–FR distance (mm)	11.70 ± 2.44	11.55 [9.85–13.04]	6.58	18.28	11.64 ± 2.73	11.76 ± 2.17	0.6304
FS–GSPN distance (mm)	5.47 ± 1.39	5.40 [4.81–6.35]	1.96	8.63	5.72 ± 1.44	5.22 ± 1.33	0.7284
FO–GSPN distance (mm)	5.26 ± 1.50	5.19 [4.23–6.32]	1.93	8.41	5.48 ± 1.46	5.04 ± 1.54	0.9687
GSPN length (mm)	10.66 ± 2.44	10.67 [8.86–12.51]	6.01	16.80	10.55 ± 2.42	10.78 ± 2.51	0.4446
GSPN–V hiatus distance (mm)	2.14 ± 1.09	2.05 [1.54–2.73]	0.00	4.25	2.06 ± 1.03	2.23 ± 1.16	0.2356
Trigeminal nerve length (mm)	10.37 ± 2.00	10.69 [8.81–11.34]	5.14	14.08	10.28 ± 1.88	10.46 ± 2.15	0.4037
AE length (mm)	10.12 ± 2.54	9.35 [8.54–12.27]	5.04	15.13	10.57 ± 2.22	9.65 ± 2.84	0.1903
V–AE distance (mm)	21.20 ± 3.69	20.60 [18.70–23.42]	12.32	28.90	21.23 ± 3.11	21.17 ± 4.31	0.5311
GSPN–ICA distance (mm)	1.84 ± 1.52	1.70 [0.52–2.75]	0.00	5.65	1.80 ± 1.78	1.88 ± 1.26	0.0134*
V–IAM (IAC) distance (mm)	7.45 ± 2.10	7.81 [6.24–8.45]	2.00	11.95	7.55 ± 1.84	7.35 ± 2.37	0.4569
IAM–SSC distance (mm)	7.02 ± 2.14	6.79 [5.51–8.82]	3.22	12.10	6.99 ± 1.97	7.06 ± 2.34	0.5466
Cochlea–GG distance (mm)	3.39 ± 0.98	3.38 [2.70–4.12]	1.14	5.52	3.59 ± 1.11	3.20 ± 0.82	0.9806
Cochlea–GSPN distance (mm)	11.69 ± 1.81	11.20 [10.37–12.74]	9.06	15.97	11.63 ± 1.68	11.74 ± 1.96	0.0148*
Cochlea–ICA genu distance (mm)	4.56 ± 1.29	4.64 [3.82–5.35]	0.65	7.39	4.51 ± 1.24	4.62 ± 1.37	0.0770
Cochlea–SSC distance (mm)	10.19 ± 1.36	10.21 [9.48–11.20]	6.62	13.38	10.05 ± 1.29	10.32 ± 1.44	0.1260
GG–GSPN (True GSPN) length (mm)	13.78 ± 1.78	13.39 [12.39–15.17]	10.86	18.28	13.84 ± 1.79	13.71 ± 1.81	0.0352*
GG–SSC distance (mm)	8.82 ± 1.49	8.86 [8.14–9.49]	5.88	13.68	9.16 ± 1.46	8.47 ± 1.48	0.1953
ICA horizontal length (mm)	6.66 ± 1.68	6.26 [5.33–7.67]	3.55	11.29	6.78 ± 1.50	6.54 ± 1.87	0.0173*
V2 intracranial length (mm)	10.61 ± 1.19	10.51 [9.77–11.42]	8.71	13.12	10.76 ± 1.22	10.45 ± 1.20	0.9437
V3 intracranial length (mm)	4.99 ± 1.08	4.93 [4.42–5.34]	2.53	7.15	4.96 ± 1.27	5.03 ± 0.90	0.7750

Data are presented as mean ± SD, median [IQR], and range; analyses were based on available data without imputation. GSPN, greater superficial petrosal nerve; AE, arcuate eminence; IAC, internal acoustic canal; SSC, superior semicircular canal; ICA, internal carotid artery; GG, geniculate ganglion.

*Significant at *p* < 0.05.

### Angular measurements

Angular relationships among key anatomical landmarks are summarized in Table 4. The GSPN–AE angle averaged 121.82 ± 15.38° (median 122.50°; range: 90.36–151.13°; *n* = 37 sides with data). The GSPN–SSC angle was 104.07 ± 13.25° (median 107.94°), and the GSPN–IAC angle was 61.37 ± 10.75° (median 60.69°). The IAC–SSC angle was 45.42 ± 12.09° (median 48.72°; range: 14.53–65.41°). Among the angular parameters, a statistically significant right–left difference was observed for the GSPN–SSC angle, which was slightly greater on the right than on the left (105.03 ± 12.97° vs. 103.12 ± 13.78°, *p* = 0.0138). No significant side differences were found for the GSPN–AE, GSPN–IAC, or IAC–SSC angles. Wide standard deviations across all angular parameters reflect substantial interindividual variability.

**Table 4:** Angular morphometric measurements of key middle cranial fossa landmarks, stratified by side (degrees).

Measurement	Total (*N* = 42)				Right (*N* = 21)	Left (*N* = 21)	*p*-value
	Mean ± SD	Median [IQR]	Min	Max	Mean ± SD	Mean ± SD	
GSPN–AE angle (°)	121.82 ± 15.38	122.50 [113.52–135.18]	90.36	151.13	124.89 ± 15.42	118.58 ± 15.08	0.8209
GSPN–SSC angle (°)	104.07 ± 13.25	107.94 [95.64–116.19]	79.26	122.59	105.03 ± 12.97	103.12 ± 13.78	0.0138*
GSPN–IAC angle (°)	61.37 ± 10.75	60.69 [52.94–67.33]	42.44	95.18	59.71 ± 8.55	63.04 ± 12.57	0.2340
IAC–SSC angle (°)	45.42 ± 12.09	48.72 [37.48–55.95]	14.53	65.41	48.29 ± 10.78	42.55 ± 12.89	0.0709

Data are presented as mean ± SD, median [IQR], and range. GSPN, greater superficial petrosal nerve; AE, arcuate eminence; SSC, superior semicircular canal; IAC, internal acoustic canal.

*Significant at *p* < 0.05.

### Sex-stratified analysis

As was shown in Table 5, sex-stratified comparisons revealed three morphometric parameters significantly larger in males than females, consistent with overall cranial size scaling rather than localized anatomical rearrangement. The cochlea–SSC distance was significantly greater in males (10.59 ± 0.70 mm vs. 9.53 ± 0.70 mm; *p* = 0.0033). The trigeminal nerve–AE distance was also greater in males (22.31 ± 2.80 mm vs. 19.07 ± 2.81 mm; *p* = 0.0238), as was the trigeminal nerve–IAC distance (8.14 ± 1.27 mm vs. 6.33 ± 2.17 mm; *p* = 0.0250). These differences reached statistical significance (*p* < 0.05) and reflect proportional scaling rather than focal anatomical variation. All other measurements showed no statistically significant differences between sexes (all *p* ≥ 0.05).

**Table 5:** Sex-stratified comparison of morphometric measurements of middle cranial fossa landmarks (mm).

Measurement	Male	Female	*p*-value
	Mean ± SD	Mean ± SD	
ZGR–FS distance (mm)	29.87 ± 1.41	29.38 ± 2.37	0.5501
FS–FO distance (mm)	3.11 ± 0.69	2.98 ± 0.63	0.6643
FO–FR distance (mm)	12.25 ± 2.47	10.81 ± 1.80	0.1709
FS–GSPN distance (mm)	5.40 ± 1.28	5.59 ± 0.78	0.7157
FO–GSPN distance (mm)	5.14 ± 1.59	5.45 ± 0.75	0.6132
GSPN length (mm)	11.21 ± 2.23	9.77 ± 1.86	0.1441
GSPN–V hiatus distance (mm)	2.14 ± 1.14	2.16 ± 0.86	0.9679
Trigeminal nerve length (mm)	10.64 ± 1.72	9.93 ± 1.35	0.3329
AE length (mm)	10.39 ± 1.81	9.89 ± 2.17	0.5920
V–AE distance (mm)	22.31 ± 2.80	19.07 ± 2.81	0.0238*
GSPN–ICA distance (mm)	2.12 ± 1.34	1.38 ± 1.17	0.2167
V–IAM (IAC) distance (mm)	8.14 ± 1.27	6.33 ± 2.17	0.0250*
IAM–SSC distance (mm)	7.46 ± 1.41	6.32 ± 2.00	0.1425
Cochlea–GG distance (mm)	3.53 ± 0.79	3.17 ± 0.91	0.3427
Cochlea–GSPN distance (mm)	12.20 ± 1.77	10.86 ± 1.08	0.0698
Cochlea–ICA genu distance (mm)	4.52 ± 0.93	4.64 ± 0.82	0.7577
Cochlea–SSC distance (mm)	10.59 ± 0.70	9.53 ± 0.70	0.0033*
GG–GSPN (True GSPN) length (mm)	14.23 ± 1.83	13.04 ± 1.15	0.1150
GG–SSC distance (mm)	9.15 ± 0.83	8.28 ± 1.10	0.0541
ICA horizontal length (mm)	7.13 ± 1.74	5.90 ± 0.74	0.0753
GSPN–AE angle (°)	121.00 ± 12.09	124.48 ± 13.11	0.7573
GSPN–SSC angle (°)	106.31 ± 12.77	100.44 ± 9.67	0.2793
GSPN–IAC angle (°)	60.91 ± 8.30	62.13 ± 6.97	0.7334
IAC–SSC angle (°)	47.00 ± 11.29	42.86 ± 9.61	0.2381
V2 intracranial length (mm)	10.57 ± 1.18	10.68 ± 0.01	0.9053
V3 intracranial length (mm)	5.15 ± 0.80	4.42 ± 0.19	0.2483

Data are presented as mean ± SD. Comparisons were performed using independent-samples *t*-tests. AE, arcuate eminence; IAC, internal acoustic canal; GSPN, greater superficial petrosal nerve; SSC, superior semicircular canal.

*Significant at *p* < 0.05.

### Bilateral (right–left) comparison

Paired right–left comparisons demonstrated overall bilateral symmetry across all 26 assessed parameters. No statistically significant side difference was observed in any linear measurement (all *p* > 0.05). Among angular parameters, one significant asymmetry was identified: the IAC–SSC angle was significantly larger on the right than the left side (48.29 ± 10.78° vs. 42.55 ± 12.89°; *p* = 0.022, paired t-test), based on all 21 paired specimens. No significant right–left differences were detected for the remaining angular measurements, including the GSPN–AE angle (*p* = 0.2044), GSPN–SSC angle (*p* = 0.4965), and GSPN–IAC angle (*p* = 0.3236). Taken together, these findings indicate that bilateral morphometric symmetry was preserved for most parameters, with the IAC–SSC angle as the only variable showing a statistically significant side difference (*p* < 0.05) (Table 6).

**Table 6:** Paired right–left comparison of linear and angular morphometric measurements.

Measurement	Right	Left	*p*-value
	Mean ± SD	Mean ± SD	
ZGR–FS distance (mm)	29.38 ± 1.76	29.99 ± 2.16	0.0983
FS–FO distance (mm)	3.30 ± 1.01	2.88 ± 0.82	0.1634
FO–FR distance (mm)	11.64 ± 2.73	11.76 ± 2.17	0.7617
FS–GSPN distance (mm)	5.72 ± 1.44	5.22 ± 1.33	0.1888
FO–GSPN distance (mm)	5.48 ± 1.46	5.04 ± 1.54	0.1776
GSPN length (mm)	10.55 ± 2.42	10.78 ± 2.51	0.6530
GSPN–V hiatus distance (mm)	2.06 ± 1.03	2.23 ± 1.16	0.5315
Trigeminal nerve length (mm)	10.28 ± 1.88	10.46 ± 2.15	0.7523
AE length (mm)	10.31 ± 2.21	9.70 ± 2.91	0.5010
V–AE distance (mm)	21.12 ± 3.08	21.35 ± 4.38	0.8261
GSPN–ICA distance (mm)	1.80 ± 1.78	1.88 ± 1.26	0.8361
V–IAM (IAC) distance (mm)	7.55 ± 1.84	7.35 ± 2.37	0.6639
IAM–SSC distance (mm)	6.99 ± 1.97	7.06 ± 2.34	0.8988
Cochlea–GG distance (mm)	3.59 ± 1.11	3.20 ± 0.82	0.0934
Cochlea–GSPN distance (mm)	11.63 ± 1.68	11.74 ± 1.96	0.7384
Cochlea–ICA genu distance (mm)	4.51 ± 1.24	4.62 ± 1.37	1.0000
Cochlea–SSC distance (mm)	10.05 ± 1.29	10.32 ± 1.44	0.5580
GG–GSPN (True GSPN) length (mm)	13.84 ± 1.79	13.71 ± 1.81	0.6726
GG–SSC distance (mm)	9.16 ± 1.46	8.47 ± 1.48	0.2157
ICA horizontal length (mm)	6.78 ± 1.50	6.54 ± 1.87	0.4544
GSPN–AE angle (°)	124.38 ± 15.89	118.35 ± 15.51	0.2044
GSPN–SSC angle (°)	105.03 ± 12.97	103.12 ± 13.78	0.4965
GSPN–IAC angle (°)	59.71 ± 8.55	63.04 ± 12.57	0.3236
IAC–SSC angle (°)	48.29 ± 10.78	42.55 ± 12.89	0.0220*
V2 intracranial length (mm)	10.81 ± 1.28	10.45 ± 1.20	0.3814
V3 intracranial length (mm)	4.91 ± 1.34	5.03 ± 0.90	0.8358

Data are presented as mean ± SD. Paired t-test was used unless otherwise specified. *p* < 0.05. IAC, internal acoustic canal; SSC, superior semicircular canal; GSPN, greater superficial petrosal nerve; AE, arcuate eminence.

*Significant at *p* < 0.05.

## Discussion

To the best of our knowledge, this study represents the first cadaveric morphometric investigation of the middle cranial fossa via the anterior petrosal approach conducted in Vietnam, based exclusively on Vietnamese specimens. By providing a systematic bilateral analysis of linear and angular relationships among key neurovascular and osseous landmarks, our findings add population-specific quantitative data to a body of literature that has largely been derived from Western or mixed cohorts. Given the known variability in skull base morphology across populations, such data are clinically relevant for surgical planning and risk mitigation in skull base surgery. In line with the revised Introduction, our results are interpreted within a structured anatomical framework centered on the Kawase rhomboid, rather than focusing on isolated landmarks. Our study also confirms significant individual variability in the mutual relationships of different surgical landmarks, echoing the findings of Maina et al. (3), who emphasized that the location of the IAC is the keystone of bone removal (3).

The present results reaffirm the GSPN as a consistently identifiable and surgically reliable landmark within the middle cranial fossa. Its constant identification rate and measurable spatial relationships to the foramen spinosum, foramen ovale, cochlea, and petrous internal carotid artery (pICA) support its central role in defining the lateral boundary of the Kawase corridor. Within the context of Kawase's rhomboid, the GSPN provides a stable lateral reference that helps delineate the safe limits of extradural drilling. In our cohort, the mean distance from the GSPN to the pICA was 1.84 ± 1.52 mm, which is a critical margin to prevent vascular injury during bone removal. This is supported by 3D-CT anatomical analyses showing that the mean shortest and longest distances to the internal auditory meatus (IAM) from the petrous ridge are approximately 5.22 mm and 10.1 mm, respectively (16). However, it is vital to note that the geniculate ganglion, which the GSPN originates from, can be dehiscent on the temporal floor in up to 16% of cases, necessitating extreme caution during initial dural elevation (4). Furthermore, the internal carotid artery itself may lack a bony covering in approximately 20% of specimens, increasing the risk of iatrogenic injury during bone removal (1, 4). To navigate this complex organization, Tawfik-Helika et al. (15) proposed a new compartmental approach, grouping contents into mucosal, cutaneous, neural, and vascular compartments to simplify the anatomy of the petrous pyramid (15). This finding is consistent with observations that the GSPN runs nearly parallel to the pICA, serving as a stable axis for identification (12). This is supported by radiological findings that a safe zone consistently exists between drilling edges and the carotid artery, though this margin can be influenced by tumor size (17). Furthermore, while our drilling window followed standard Kawase boundaries, quantitative data suggest that medial mobilization of the trigeminal nerve—as seen in the modified Dolenc-Kawase approach—can increase the surgical area by up to 104% compared to the traditional window (18). Importantly, the quantitative ranges reported in our Vietnamese cohort provide additional reference values that may assist surgeons in anticipating safe drilling margins, particularly in anatomically compact skull bases.

Our linear morphometric data further delineate the spatial configuration of the Kawase drilling window as a three-dimensional construct rather than a simple planar triangle. Distances among foramina, trigeminal nerve segments, and cochlear landmarks demonstrate measurable variability yet preserved central tendencies across specimens. These findings support the concept that the Kawase corridor should be understood as an integrated spatial framework, rather than as a collection of independent anatomical measurements. The volumetric analysis of the petrous apex outlined by the Kawase triangle in our study can be compared to the findings of Pérez et al. (5), who reported an average volume of 1.89 ± 0.52 cm^3^, noting that such measurements are vital for quantifying the safe limits of anterior petrosectomy (5). The petrous apex, which forms the core of this corridor, is a complex anatomical “crossroad” where the internal carotid artery, the trigeminal nerve, and the abducens nerve converge (2). While our study focused on standard osseous landmarks, other techniques have sought to simplify IAC localization using external references. For example, the zygomatic root has been used to identify the malleus head, which lies in a straight line with the IAC, approximately 18 mm medial to the temporal squamosa (11). This external reference can be further supplemented by using the middle point of the external auditory canal (MEAC), which typically lies 16 mm posterior and 24 mm lateral to the petrous apex (19). Advanced imaging atlases also emphasize that the porus acusticus of the IAC serves as a critical medial reference, situated superior to the cochlear aqueduct and anterior to the vestibular aqueduct (20). Additionally, the line connecting the arcuate eminence to the trigeminal notch has been proposed as a direct projection of the anterior wall of the IAC, measuring 18.25 ± 2.20 mm in Chinese specimens (8).

Angular measurements provide additional insight into the three-dimensional orientation of critical landmarks encountered during anterior petrosectomy. The angular relationships between the GSPN, arcuate eminence (AE), superior semicircular canal, and IAC highlight both predictable spatial patterns and notable inter-individual variability. Our findings of significant variability in the GSPN-AE angle (121.82 ± 15.38°) reinforce the observation that surface landmarks can be unreliable. This aligns with the conclusion that surgeons should use preoperative 3D-CT to determine patient-specific distances and angles between critical structures (16). Maina et al. (3) also noted a smaller surgical window in female patients, a factor that can be predicted on preoperative imaging to avoid complications (3). Cheng reported that the AE is unidentifiable in 15% of cases, suggesting that the intersection of the trigeminal ganglion and superior petrosal sinus (Point T) can serve as a more stable alternative, with the IAC located by tracing 1 cm posteriorly from Point T and turning laterally 135° (21). The localization of the IAC remains the most critical and challenging step; early foundational studies established that the IAC is typically situated at a mean distance of 2.8 mm from the geniculate ganglion and 10.1 mm from the AE (22). Furthermore, recent findings suggest that angles based on the foramen ovale and foramen spinosum (FO-FS-IAM angle) exhibit lower variance than those based on the AE, making them potentially more effective for meatus localization (9). Surgeons can also employ the “Rule of 2s”, where the distance from the foramen spinosum to the IAC is approximately 2 cm, to verify orientation when other markers are featureless (13). These findings further support the integration of quantitative morphometric data with neuronavigation systems to enhance surgical precision. Despite these advancements, the use of neuronavigation systems alongside detailed neuroanatomic knowledge remains essential to compensate for the featureless nature of the middle cranial base and ensure a safe procedure (6).

Bilateral comparison revealed a high degree of right–left symmetry for most measured parameters, with only limited statistically significant differences. This finding aligns with earlier morphometric studies of the middle cranial fossa and petrous apex, which generally report symmetrical anatomy with occasional localized asymmetry. In the present series, no significant right–left differences were found for linear measurements on paired analysis, whereas among angular parameters, only the IAC–SSC angle showed a significant side difference, being greater on the right than on the left (48.29 ± 10.78° vs. 42.55 ± 12.89°; *p* = 0.0220). From a surgical perspective, such symmetry supports the use of contralateral anatomy as a reference during unilateral pathology, while underscoring the importance of respecting individual variations—particularly in the relationship between the IAC and the superior semicircular canal. Mastering these variations is essential not only for safe drilling but also for effectively managing tegmen repairs and complex tumor resections (23). Moreover, defining a “safe area of intradural petrosectomy” is paramount to prevent catastrophic injury to the labyrinthine structures and venous sinuses (24). To further refine this “minefield”, the use of an “intertriangles line” has been proposed to divide the Kawase area into safer zones, protecting the cochlea during bone removal (14).

The population specificity of this study warrants particular emphasis. Prior investigations have demonstrated that cranial base morphology can vary according to ethnicity and geographic origin, potentially influencing surgical exposure and complication profiles. By focusing exclusively on Vietnamese cadavers, our study provides anatomically relevant data for Southeast Asian patients and contributes to the growing recognition that region-specific anatomical research is essential for refining skull base surgical strategies. These data may also inform future comparative studies, surgical simulation models, and integration into navigation-assisted surgical planning frameworks.

Several limitations of this study should be acknowledged. First, as a cadaveric investigation, the findings may not fully replicate intraoperative conditions in living patients, particularly with respect to tissue elasticity and vascular dynamics. Second, although the sample size is comparable to that of many detailed skull base anatomical studies, it may not capture the entire spectrum of anatomical variability within the Vietnamese population. Third, all dissections and measurements were performed by a single neurosurgeon; while this minimized inter-observer variability, it precluded formal assessment of inter-rater reliability. Finally, some measurements could not be obtained in all specimens due to anatomical variation or preservation-related factors, reflecting true anatomical inaccessibility rather than methodological omission, resulting in missing data that were intentionally not imputed. Despite these limitations, the present study provides robust, systematically acquired morphometric data that strengthen the anatomical foundation of the anterior petrosal approach in a previously unstudied population.

## Conclusions

To our knowledge, this study represents one of the first cadaveric morphometric investigations of the middle cranial fossa via the anterior petrosal approach conducted in Vietnam, based exclusively on Vietnamese specimens. By providing a systematic bilateral analysis of linear and angular relationships among key neurovascular and osseous landmarks, our findings add population-specific quantitative data to a body of literature that has largely been derived from Western or mixed cohorts. Most measurements showed bilateral symmetry, with only limited side-specific differences. Given the known variability in skull base morphology across populations, such data are clinically relevant for surgical planning and risk mitigation, and may support the development of navigation-assisted techniques and training applications in skull base surgery.

## Data Availability

The raw data supporting the conclusions of this article will be made available by the authors, without undue reservation.
